# Sex‐specific effects of outbreeding on offspring quality in pike (*Esox lucius*)

**DOI:** 10.1002/ece3.4510

**Published:** 2018-10-02

**Authors:** Johanna Sunde, Petter Tibblin, Per Larsson, Anders Forsman

**Affiliations:** ^1^ Department of Biology and Environmental Science Ecology and Evolution in Microbial Model Systems, EEMiS Linnaeus University Kalmar Sweden

**Keywords:** *Esox lucius*, genetic admixture, interbreeding, offspring quality, outbreeding, reproductive performance

## Abstract

Intraspecific genetic admixture occurs when previously separated populations within a species start interbreeding, and it can have either positive, negative, or neutral effects on reproductive performance. As there currently is no reliable predictor for the outcome of admixture, an increased knowledge about admixture effects in different species and populations is important to increase the understanding about what determines the response to admixture. We tested for effects of admixture on F1 offspring quality in three subpopulations of pike (*Esox lucius*). Gametes were collected in the field, and eggs from each female were experimentally fertilized with milt from a male from each population (one “pure” and two “admixed” treatments). Three offspring quality measures (hatching success, fry survival, and fry length) were determined and compared between (a) pure and admixed population combinations and (b) the sex‐specific treatments within each admixed population combination (based on the origin of the male and female, respectively). The results suggested that although there were no overall effects of admixture on offspring quality, the consequences for a given population combination could be sex‐specific and thus differ depending on which of the parents originated from one or the other population. All offspring quality traits were influenced by both maternal ID and paternal ID. Sex‐ and individual‐specific effects can have implications for dispersal behavior and gene flow between natural populations, and are important to consider in conservation efforts.

## 1 INTRODUCTION

Intraspecific genetic admixture, or interpopulation hybridization, occurs when previously separated populations start interbreeding (Lynch, 1991). Both natural dispersal and human‐mediated translocations (intentional and accidental) can result in such admixture (Verhoeven, Macel, Wolfe, & Biere, 2011), and it can have positive, negative, or neutral fitness effects on populations and individuals (Lynch, 1991; McClelland & Naish, 2007; Rollinson et al., 2014; Simberloff, 2009; Sunde & Forsman, 2016; Tinnert, Berggren, & Forsman, 2016; Verhoeven et al., 2011; Weeks et al., 2011; Whitlock et al., 2013).

If populations are genetically divergent, admixture will increase the genetic diversity in the receiving population (Harpur, Minaei, Kent, & Zayed, 2012), which can prevent or counteract inbreeding depression, and conceal deleterious recessive alleles (Keller & Waller, 2002; Lynch, 1991; Weeks et al., 2011). Admixture may also enable creation of novel genotypes and haplotypes, and can result in heterosis (Drake, 2006; Facon, Jarne, Pointier, & David, 2005; Fenster & Galloway, 2000a; Lavergne & Molofsky, 2007; Lynch, 1991). Admixture can thus be beneficial because genetically and phenotypically more diverse populations are more resilient, better able to adapt to environmental changes, and have been shown to have higher colonization success than less diverse populations (Forsman, 2014; Forsman & Wennersten, 2016; Rieseberg, Archer, & Wayne, 1999; Rius & Darling, 2014).

On the contrary, admixture can also have detrimental fitness effects by diluting favorable alleles, and by the breaking of coadapted gene complexes resulting from local adaptations (Edmands, 2007; Fenster & Galloway, 2000a; Lynch, 1991; Rhymer & Simberloff, 1996; Verhoeven et al., 2011; Whitlock et al., 2013). Additionally, admixture can impair fertility and viability in the offspring (Gilk et al., 2004; Sunde & Forsman, 2016; Turner, Schwahn, & Harr, 2012).

The net outcome of genetic admixture is determined by all these interactive mechanisms, and by the inherent properties (e.g., genetic architecture and local adaptations) of the populations involved. The magnitude and direction of the outcome can therefore vary between species (Hardiman & Culley, 2010; Molofsky, Keller, Lavergne, Kaproth, & Eppinga, 2014; Rollinson et al., 2014) and differ among populations within species (Escobar, Nicot, & David, 2008; Hufford, Krauss, & Veneklaas, 2012; Sunde & Forsman, 2016; Tinnert et al., 2016; Tortajada, Carmona, & Serra, 2010). Such intraspecific asymmetric responses to admixture have potential to affect the genetic diversity and architecture within populations as well as divergence between populations.

Unravelling the roles of admixture is potentially complicated further by sex‐specific responses. Males and females resolve life history tradeoffs differently and are often subjected to opposing (antagonistic) selection, leading to sex‐specific genetic variation and sex‐specific genetic architecture of life history traits (Forsman, 1995, 2018; Lande, 1980; Rice, 1984; Roff, 1992; Trivers, 1972; Zwoinska, Lind, Cortazar‐Chinarro, Ramsden, & Maklakov, 2016). The consequences of genetic admixture, how response patterns might vary among populations within species and according to sex of immigrants within populations must be further investigated to increase the understanding of what determines the outcome of admixture, to search for any general response patterns, and to facilitate successful management (Rius & Darling, 2014).

**Figure 1:**
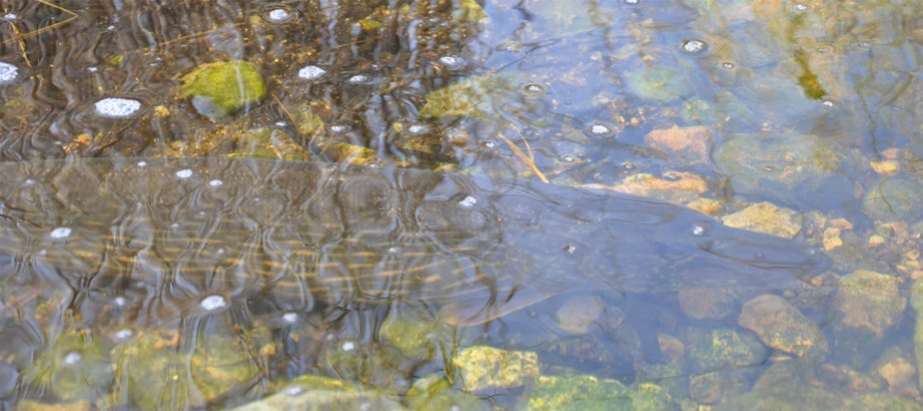
An adult pike (*Esox lucius*) in the water by the inlet to one of the study localities (Harfjärden). Photograph: Anders Forsman

The present study investigates the effects of intraspecific genetic admixture on different aspects of F1 offspring quality in anadromous subpopulations of pike (*Esox lucius*) (Figure 1). Pike is a long‐lived, iteroparous, top‐predatory fish, with a circumpolar distribution, and an important model species in studies of ecology and evolution (Forsman et al., 2015). Moreover, pike is a highly valued species for recreational and commercial fishing (Lehtonen, Leskinen, Selen, & Reinikainen, 2009; Pierce, Tomcko, & Schupp, 1995) and subjected to large‐scale stocking programs throughout its distribution (Craig, 1996; Larsen, Hansen, Nielsen, Jensen, & Loeschcke, 2005; Skov & Nilsson, 2007).

**Figure 2:**
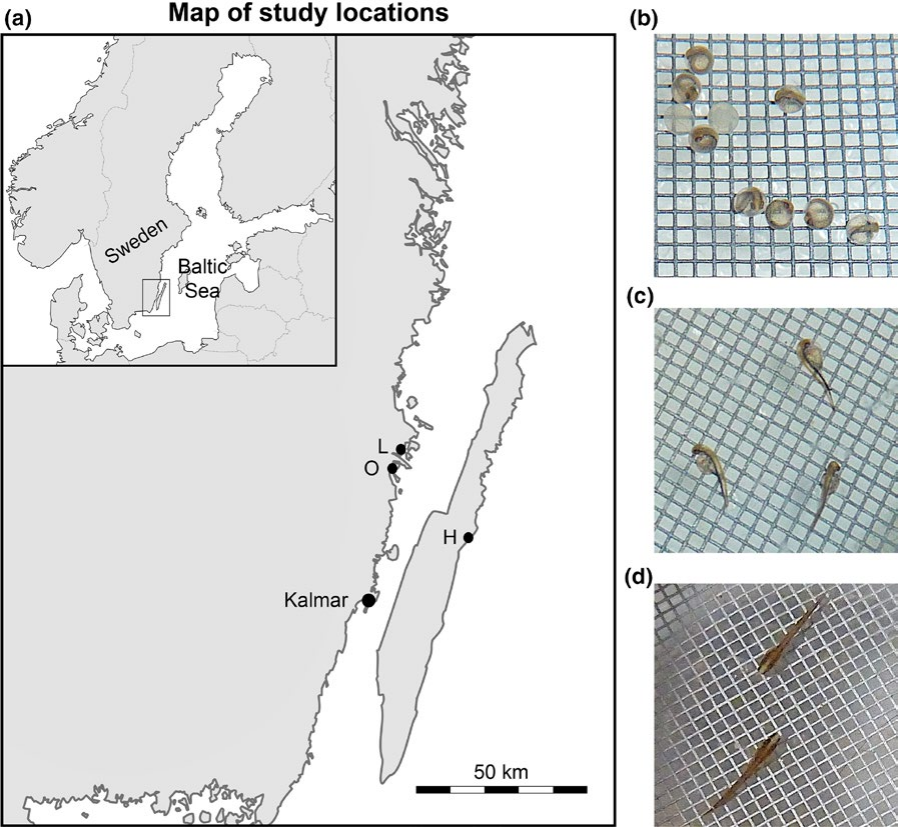
Study area and early development of pike (*Esox lucius*). (a) a map of the study locations, Lerviksbäcken (L), Oknebäcken (O) and Harfjärden (H). The map was generated in Adobe Photoshop CC, version 2015.0.1. The figure was modified from two base maps, one of Scandinavia and one of Sweden, which are available under nonrestrictive creative commons license obtained from Wikimedia Commons, https://commons.wikimedia.org/wiki/File:Scandinavia-template.png and https://commons.wikimedia.org/wiki/File:Sweden_location_map.svg. Photographs show: (b) eggs with developing embryos at day 7, (c) newly hatched yolk‐sac larvae at day 9, and (d) surviving fry at day 23

In this study, pike from three subpopulations (for sampling locations see Figure 2a) that display different local adaptations (e.g., larval traits (Berggren, Nordahl, Tibblin, Larsson, & Forsman, 2016), body size and growth rate (Tibblin et al., 2015), vertebral number (Tibblin, Berggren, Nordahl, Larsson, & Forsman, 2016), and salinity tolerance (Sunde, Tamario, Tibblin, Larsson, & Forsman, 2018)) were used in a common garden admixture experiment. Three offspring quality traits (hatching success, fry survival, and fry length) were measured and compared between (a) population combinations (“population combination comparisons”) and (b) the two treatments within each population combination, based on the origin of the male and female, respectively (“sex‐separated comparisons”). The population combination comparisons, where both treatments from a population combination were pooled, can be considered to primarily mimic the movement of a group of individuals of mixed sexes (e.g., management efforts such as supplementations and translocations of a group of individuals of mixed sexes). The sex‐separated admixture comparisons on the other hand, primarily mimic the effects that would be expected following immigration of a single individual (e.g., natural dispersal), or of a group of individuals of the same sex.

The main objectives were to investigate whether (a) there were any effects of genetic admixture on F1 offspring quality, (b) the magnitude and/or direction of admixture effects varied between populations and population combinations, and (c) the consequences of interbreeding were sex‐specific and varied depending on the source population of the male and female, respectively.

## 2 MATERIAL AND METHODS

### 2.1 Study species

In the Kalmar sound area of the Baltic Sea, where pikes were sampled for this study (Figure 2a), anadromous subpopulations of pike show a strong homing behavior (Engstedt, Engkvist, & Larsson, 2014; Larsson et al., 2015; Tibblin, Forsman, Borger, & Larsson, 2016), returning to their natal freshwater habitats (e.g., streams and wetlands) to spawn (Engstedt, Stenroth, Larsson, Ljunggren, & Elfman, 2010; Engstedt et al., 2014; Muller, 1986). This homing behavior entails reproductive isolation between populations, which has allowed even closely located populations to become genetically differentiated (Larsson et al., 2015; Tibblin et al., 2015).

Estimates of population genetic diversity and differentiation based on neutral molecular markers (such as microsatellites) do not reliably inform about functional genetic variation and local adaptations (Hedrick, 2001; Holderegger, Kamm, & Gugerli, 2006; Leinonen, O'Hara, Cano, & Merilä, 2008; Reed & Frankham, 2001). However, previous studies have also found evidence of local adaptations in larval traits (Berggren et al., 2016), body size and growth rate (Tibblin et al., 2015), vertebral number (Tibblin, Berggren, et al., 2016), and salinity tolerance (Sunde et al., 2018) between adjacent, anadromous subpopulations of pike in the Baltic Sea. The fine‐scaled genetic structuring and the presence of local adaptations suggest that both inbreeding and outbreeding are potentially important in pike, possibly affecting offspring quality and population viability. This, in combination with the fact that pike has external fertilization and can be reared in common garden environments makes it amenable for experimental studies of interpopulation genetic admixture.

### 2.2 Study localities and sampling procedure

The three subpopulations of pike used in the present study (Harfjärden, Lerviksbäcken and Oknebäck; henceforth Harfjärden, Lervik and Okne) spawn in the Southeastern part of Sweden (Figure 2a). Two of the populations spawn in adjacent wetlands in the southeast of the Swedish mainland (Lervik: N57° 04.414′; E16° 31.246′, and Okne: N57° 01.200′; E16° 26.700′), and the third population spawns in a wetland on the east coast of the island of Öland (Harfjärden: N56° 49.063′; E16° 48.673′). Thus, the geographic distance between the locations differ (shortest waterway distance: Okne–Lervik: 20 km; Harfjärden–Lervik: 120 km; Harfjärden–Okne: 135 km, for sampling locations see Figure 2a). Nevertheless, all three subpopulations are genetically differentiated from each other (Pairwise *F*_ST_‐values: Okne–Lervik: 0.044; Harfjärden–Okne: 0.212; Harfjärden–Lervik: 0.226; *p *<* *0.05), which was revealed in the study by Larsson et al. (2015), where genetic variation was analyzed based on 10 microsatellite loci (Elu 2 and Elu 6 [Hansen, Taggart, & Meldrup, 1999], Elu 19, Elu 37, Elu 51, Elu 64, Elu 76, Elu 78, Elu 87 and Elu 276 [Miller & Kapuscinski, 1996, 1997]).

Adult pikes from each subpopulation were caught during 4 days (March 31, April 1, April 4, and April 5, 2016) using fyke nets that were placed in the inlet of the streams leading to the spawning grounds (Sunde et al., 2018; Tibblin, Berggren, et al., 2016), and ripe individuals (Harfjärden: 11 females, 12 males; Lervik: 10 females, 12 males; Okne: 11 females, 10 males) were stripped of gametes. Stripping was synchronized between the localities to avoid systematic error due to different storing times of gametes before artificial fertilization was conducted. To ensure high‐quality eggs, only females with eggs that had no visual trace of blood were selected, and the first batch of eggs from each selected female was discarded to avoid premature fertilization activation of the eggs (opening of the micropyle) (Craig, 1996; Raat, 1988). The gametes were collected in separate sterile plastic tubes (eggs: 50 ml Falcon tubes, milt: 2 ml Eppendorf tubes) that were immediately put on ice in cooler boxes. The tubes were continuously kept on ice (up to 4 hr) during the transportation to the Kalmarsound Laboratory of Linnaeus University in Kalmar, Sweden, until fertilization.

### 2.3 Admixture experiment

The collected gametes were used in a common garden admixture experiment. To that end, each female was paired with one male from each subpopulation, and each male was also paired with one female from each subpopulation. In general, each individual contributed gametes to only one family (female/male pair) in each population combination. However, occasional mismatches occurred due to unequal sex ratios of the captured individuals on different days. The eggs from each female were divided into six separate batches (25 eggs in each) which each was experimentally fertilized with milt from a male from one of the subpopulations (making two replicates each of one “pure” and two different “admixed” treatments per female). All families were thus duplicated, resulting in a total of approximately 4,800 eggs divided between 192 experimental units. The artificial fertilization and the entire experiment was carried out in a constant room with a temperature of 12°C, using tap water that had been aerated for a minimum of four hours. The design was chosen such that the temperature was within the natural range (Frost & Kipling, 1967; Nilsson, Engstedt, & Larsson, 2014), and because other studies have successfully used similar approaches (Sunde et al., 2018; Tibblin, Koch‐Schmidt, Larsson, & Stenroth, 2012; Tibblin et al., 2015).

The artificial fertilizations were conducted following the protocol used by Berggren et al. (2016) and Sunde et al. (2018). In short, eggs from one female were placed in a small glass bowl (50 mm diameter), and an excess of milt (approximately 200 μl) from one male was poured onto the eggs. Water (approximately an equal volume to the eggs) was subsequently added to the gametes to initiate fertilization, and the bowl was gently swirled in circular motions to mix the gametes. The gametes were let to rest for two minutes for fertilization to occur, and excess milt was then removed by rinsing the eggs with water three times. The fertilized eggs were then gently placed into separate plastic bins. Eggs and hatched fry were continuously kept in their natal bins throughout the experiment. Each bin consisted of two 800 ml plastic cups, placed in each other, with the bottom of the inner cup replaced with a plastic net (mesh size approximately 1.5 × 1.5 mm). This design of the bins enabled complete daily water exchanges throughout the experiment (including the time of egg incubation, when eggs are sensitive to movements) by addition of water to the cups.

The 192 experimental units were placed randomly with regards to treatment and source population in the constant room, to avoid systematic effects associated with differential air flow, temperature or light. To ensure that there was food available when fry reached the end of the yolk‐sac phase, an excess of live brine shrimp (*Artemia salina*) were provided three times per day starting 5 days post hatch. At 8 days post hatch, fry were also provided with an excess of *Daphnia spp*. From one‐day postfertilization, each unit was photographed daily (using Panasonic DMC‐TZ5), for subsequent data extraction. At the termination of the experiment, fry in each unit was also photographed against the cage bottom (with known mesh size, see Figure 2b–d) for subsequent measuring of fry body length from the pictures, using the software ImageJ.

### 2.4 Data collection

Data on different aspects of offspring quality were obtained from the photograph series. Offspring quality was estimated as (a) hatching success of eggs, (b) fry survival during 15 days following hatching, and (c) fry body length at 15 days post hatch (Figure 2b–d). We know from a previous study (Sunde et al., 2018) that there is a time period during which unfertilized eggs are not visibly discernible as dead, such that unsuccessful fertilization cannot be reliably distinguished from postfertilization embryonic mortality. We therefore estimate hatching success as a composite measure from eggs to successful hatchlings.

### 2.5 Analytical approach

One of the advantages with using pike as study species is that it has external fertilization, which enables utilization of eggs and milt from the same individuals in all treatments. This design allows controlling for variation in the response variables owing to any parental effects, and thus allows for improved power of the statistical evaluation of experimental treatments and fixed effects. Including all possible pairwise population combinations in the experimental design also enables comparisons between pooled population combinations (regardless of which sex in a specific combination of populations originates from which of the populations; henceforth “population combination comparison”) as well as comparisons between all pairwise population treatments separated by origin of the two sexes, respectively (henceforth “sex‐separated admixture comparison”). To evaluate the different ways by which admixture, source populations, and parental identity might affect offspring quality, data from the experiment were analyzed in two different ways. This was necessary to address different hypotheses and to analyze effects at different levels of resolution.

### 2.6 Statistical analyses

#### 2.6.1 Population combination comparisons

First, we addressed the overall questions whether and how admixture influenced offspring quality in different combinations of populations, and whether the effect of admixture differed among the populations. To that end, we used separate analyses on the three offspring quality traits and compared the performance of the six population combinations (the three populations pure, “HH,” “OO,” and “LL,” and all three hybrid population combinations, “OH,” “LO,” and “HL”). Population combination was treated as a fixed factor, and family (female/male pair) and replicate (nested in family) were treated as random factors. Different types of models were selected depending on the distribution of response variables. Data on fry body length, with a normal response distribution, were analyzed with general linear mixed model using the lme4 package (version 1.1‐15, lmer function) in RStudio 2 v1.1.383 (RStudio Team 2015), with R v.3.2.2 (R Core Team 2012). The effects of admixture on hatching success and survival of fry, both with binary response distributions, were analyzed with generalized linear mixed models (glmer function) with a binomial fit and a logit‐link function using the same R package.

#### 2.6.2 Sex‐specific comparisons

Next, we set out to answer the question whether offspring quality measures within population combinations varied depending on the source of the male and female, respectively. For that, we used similar analyses as described in the previous paragraph (general linear mixed model for fry length, and generalized linear mixed models for hatching success and survival). Maternal population (Mpop) and paternal population (Ppop) were treated as fixed factors, and the interaction between maternal population and paternal population (Mpop × Ppop) was included to evaluate whether the sex effects of admixture varied among populations. Family (female/male pair) and replicate (nested within family) were treated as random factors. Differences between sex‐separated pairwise admixed population combinations were based on comparisons of least squares means.

#### 2.6.3 Tests of statistical significance

Null hypothesis significance testing and statistical inference based on *p*‐values is controversial and can be complicated, particularly when analyses include complex models, multiple statistical tests, and when *p* is near the typical threshold 0.05 (Wasserstein & Lazar, 2016). A *p*‐value by itself does not measure the size of a difference or the importance of an effect, nor does it provide a good measure of evidence in favor or against the null hypothesis, and results must, therefore, be interpreted with caution (Wasserstein & Lazar, 2016). Yet, statistical significance is still considered important by some and may be used for meta‐analyses. We therefore adhere to the tradition yet treat *p*‐values carefully.

For both the population combination comparisons and the sex‐specific comparisons, statistical significance of fixed factors and of interactions was assessed using the Type III test of fixed effect. Models were fitted to the data using the restricted maximum likelihood method (Laplace approximation). The effects and statistical significance of parental identity were assessed using covariance parameter estimates. We do not report results associated with the replicate factor.

### 2.7 Ethics note

All applicable national guidelines for the care and use of animals were followed. The study conforms to Directive 2010/63/EU. Ethical approval for the study was granted by the Ethical Committee on Animal Research in Linköping, Sweden (approval Dnr ID 83). Permission for field studies was granted by the County Administrative Board in Kalmar (approval 623‐1681‐13).

## 3 RESULTS

The analyses revealed the following overall results: (a) No important overall effects of admixture on offspring quality measures were found in any of the hybrid population combinations. (b) The effect of admixture within hybrid population combinations could vary depending on the source of the male and female, respectively. (c) Offspring quality measures were influenced by both paternal and maternal identity. These findings are presented in higher resolution for each of the three response variables below.

### 3.1 Hatching success

**Figure 3:**
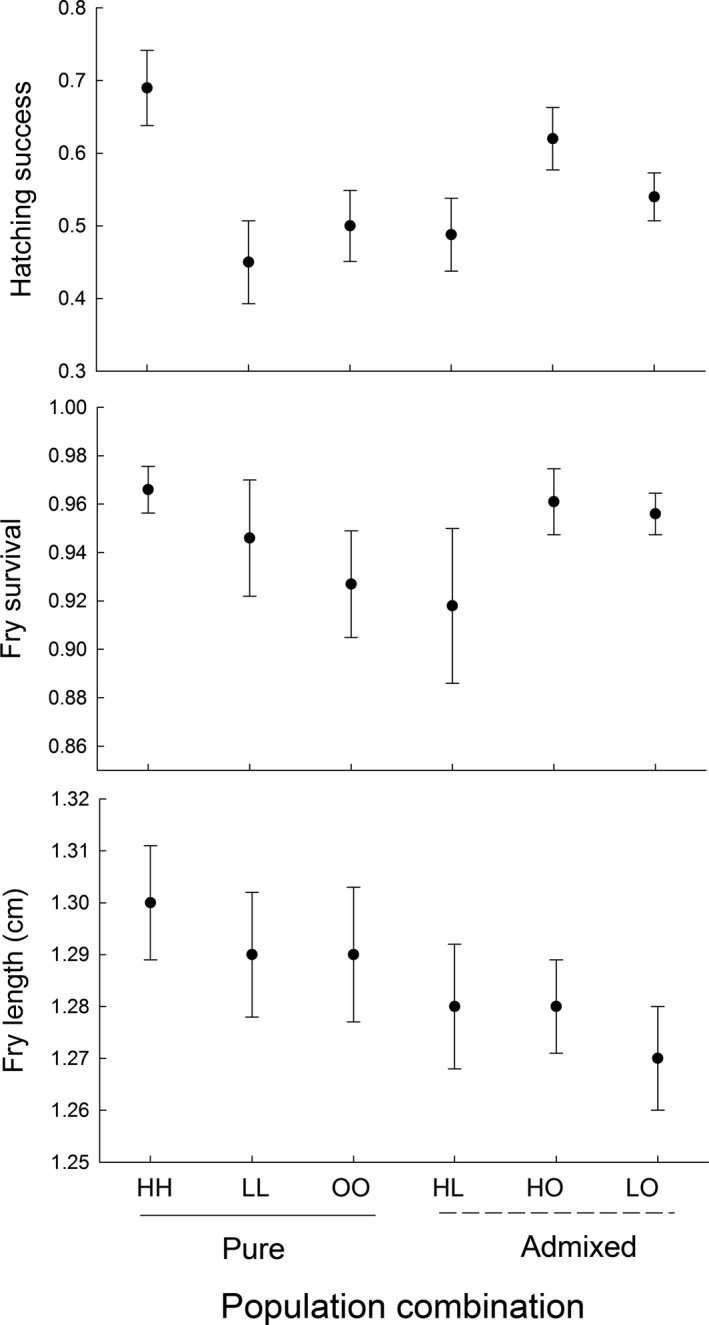
Effects of admixture and population identity on viability and performance of eggs and offspring in *Esox lucius*. Data for three populations (H = Harfjärden, L = Lervik, O = Oknebäck) that were purebred and mixed. Figure shows means ± *SE* based on raw data. Results from statistical analysis of variation among groups are presented in Table 1

Average hatching success was approximately 55% (range 43%–74%). This is comparable with estimates obtained in a previous experiment conducted in the wild (Berggren et al., 2016). The population combination comparison suggested that hatching success differed between population combinations (*p *=* *0.04, Figure 3, Table 1). However, no differences were present between the pure populations and their two respective hybrid groups. Further, the results from the sex‐separated comparison showed that hatching success was influenced by maternal populations (*p *<* *0.0001), but was not influenced by paternal population (*p *=* *0.48) or the interaction between maternal and paternal population (*p *=* *0.92; Table 2).

**Table 1:** Results from comparisons of different aspects of offspring quality among purebred (three combinations) and admixed (three combinations) experimental crossings of three natural populations of *Esox lucius*,* p*‐values < 0.05 are indicated in bold

Trait	Procedure	*F*‐value	*p*‐Value
Hatching success	*glmer*	2.34	**0.04**
Fry survival	*glmer*	0.31	0.90
Fry length	*lmer on raw data*	0.84	0.53

**Table 2:** Comparison of different aspects of offspring quality among all nine possible pairwise experimental crossings of males and females from three natural populations of *Esox lucius*,* p*‐values < 0.05 are indicated in bold

Trait	Procedure	*F*‐value	*p*‐Value
Hatching success	*glmer*		
Mpop		19.50	**<0.0001**
Ppop		0.74	0.48
Mpop × Ppop		0.24	0.92
Fry survival	*glmer*		
Mpop		1.07	0.36
Ppop		0.15	0.84
Mpop × Ppop		0.66	0.59
Fry length	*lmer on raw data*		
Mpop		3.27	**0.04**
Ppop		0.90	0.33
Mpop × Ppop		0.99	0.41

Note

Effects of maternal population (Mpop), paternal population (Ppop), and the interaction between maternal population and paternal population on hatching success, fry survival, and fry length are presented. Covariance parameter estimates associated with random effects of maternal and paternal identity are presented in Table 4.

**Figure 4:**
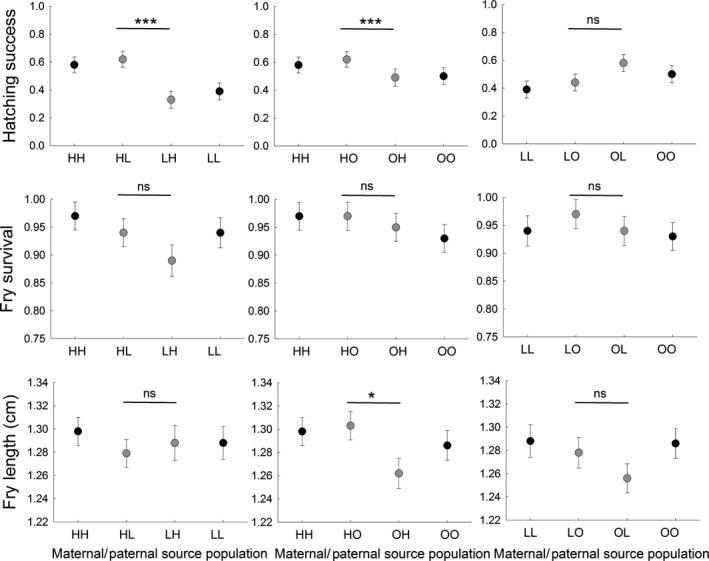
Comparisons of different aspects of offspring quality among purebred and sex‐separated admixed population combinations of *Esox lucius* from three natural populations (H = Harfjärden, L = Lervik, O = Oknebäck). Each panel shows one of the three offspring quality measures, and each plot within the panels shows a comparison between two of the pure populations (in black) and their respective sex‐separated admixed population combinations (in gray), where names indicate maternal/paternal origin. Figure shows least squares means ± *SE*. Results from the pairwise comparisons are presented in Table 3

Additionally, the least squares means comparisons showed that there were sex‐specific effects of admixture on hatching success that depended on the source populations of the male and female parent within two out of the three population combinations (HL vs. LH, *p *= <0.0001, and HO vs. OH, *p *=* *0.0004, Figure 4, Table 3). The probability associated with the third population combination (OL vs. LO) was also relatively low (*p *=* *0.095), potentially reinforcing the idea that there is a sex‐specific effect.

**Table 3:** Comparison of different aspects of offspring quality among purebred (three combinations) and sex‐separated, pairwise, admixed (six combinations) population combinations of male and female *Esox lucius* from three natural populations, *p*‐values < 0.05 are indicated in bold

Trait	Pairwise comparison	*df*	*p*‐Value
Hatching success	LH vs. HL	168.6	**<0.0001**
	OH vs. HO	163.6	**<0.001**
	OL vs. LO	156.6	0.10
Fry survival	LH vs. HL	179	0.15
	OH vs. HO	179	0.64
	OL vs. LO	179	0.27
Fry length	LH vs. HL	162	0.66
	OH vs. HO	158	**0.02**
	OL vs. LO	156	0.26

### 3.2 Fry survival

Survival of fry was high (>85%) in all groups. The population combination comparison showed that fry survival did not differ between the population combinations (*p *=* *0.90, Figure 3, Table 1). The sex‐specific comparisons further showed that neither paternal population (*p *=* *0.84), maternal population (*p *=* *0.36), nor the interaction between maternal and paternal population (*p *=* *0.59) influenced the response (Table 2). Additionally, the least squares means comparisons indicated no strong sex‐specific responses, that is the response did not seem to differ within population combinations depending on the source population of the male and female parent (*p *>* *0.05, Figure 4, Table 3).

### 3.3 Fry length

The population combination comparison showed that fry body length did not differ between population combinations (*p *=* *0.52, Figure 3, Table 1). The sex‐separated comparisons suggested that fry length varied between maternal source populations (*p *=* *0.04), but was not influenced by paternal population (*p *=* *0.33) or the interaction between maternal and paternal population (*p *=* *0.41; Table 2). The least squares means comparisons suggested that there was a sex‐specific effect of admixture on fry length within the Harfjärden—Okne population combination (HO vs. OH, *p *=* *0.02, Figure 4, Table 3).

### 3.4 Effects of maternal and paternal identity

There were moderate to strong effects of maternal identity and paternal identity on hatching success and fry survival (as evidenced by the covariance parameter estimates reported in Table 4).

**Table 4:** Effects of maternal identity (MID) and paternal identity (PID) on different aspects of offspring quality in experimental crossings of three natural populations of *Esox lucius*

Trait	Procedure	Covariance parameter estimate ± *SE*
Hatching success	*glmer*	
MID		1.2692 ± 0.1960
PID		0.0701 ± 0.0447
Fry survival	*glmer*	
MID		0.3424 ± 0.1020
PID		1.0566 ± 0.1737
Fry length	*lmer on raw data*	
MID		0.0012 ± 0.0061
PID		0.0006 ± 0.0042

Note

Random effects are indicated by covariance parameter estimates obtained using generalized linear mixed models (glmer) and general linear mixed models (lmer), respectively. The fitted statistical models included fixed explanatory variables (maternal source population, paternal source population, and the interaction between maternal and paternal source population) reported in Table 2.

## 4 DISCUSSION

Both natural dispersal and conservation efforts in the form of translocations and supplementations (also known as “stocking” in fisheries management) are likely to result in interpopulation hybridization and genetic admixture. Knowledge about how genetic admixture impacts on viability of individuals and populations and on genetic composition of natural populations is thus crucial for a better understanding of evolution of biological diversity, and for designing conservation efforts aiming at revitalizing endangered populations and protecting biodiversity. We therefore conducted an experiment with three subpopulations of anadromous pike from the Baltic Sea to investigate the effects of genetic admixture on different aspects of offspring quality.

The analytical approach in the present study included two different analyses, to do comparisons at different levels of resolution (“population combination comparison” and “sex‐separated comparison”). Results, therefore, inform about the effects of genetic admixture that might result from different types of gene flow.

### 4.1 Effects of admixture

The population combination comparison suggested that the effect of genetic admixture on survival and fry body length did not differ between any of the population combinations. Further, hatching success differed between population combinations, but no differences were found between any of the purebred populations and its two respective hybrid population combinations. This suggests that genetic admixture does not have any effect on offspring quality in the pike populations included in this study. That admixture does not substantially reduce or increase aspects of F1 offspring performance is interesting and relevant to population management. However, results from admixture studies must be interpreted with caution, especially if they will be used as guidelines for management. Several studies have found that different populations within species can respond in different ways to genetic admixture (Escobar et al., 2008; Hufford et al., 2012; Sunde & Forsman, 2016; Tinnert et al., 2016; Tortajada et al., 2010). Thus, even though the results showed that admixture did not affect offspring quality of pike in the included populations and population combinations, this outcome is not necessarily transferable to other pike populations. In addition, previous studies have found that the response to genetic admixture can differ between generations (Huff, Miller, Chizinski, & Vondracek, 2011; Tinnert et al., 2016), and between different environments (see McClelland & Naish, 2007). Thus, that no overall effects of admixture were found in this study, does not ensure that there will not be any effects under natural conditions (with different selective regimes) or in later generations (e.g., due to break‐up of coadapted gene complexes). Due to the complex nature of the interacting mechanisms determining the response to genetic admixture, it is important that the role of admixture is further studied in different species and populations to search for general patterns that might be used to predict the direction of the response (Rius & Darling, 2014).

We observed a trend that fry in admixed population combinations were smaller than fry in purebred treatments (Figure 3). Body size as a fitness estimate might be an especially important proxy in pike where cannibalism in fry is common (Craig, 1996). A large body could enable fry to prey on smaller conspecifics, and protect them from being victims of cannibalism. Previous investigations based on *Q*_ST_/*F*_ST_ comparisons and common garden breeding experiments have also indicated that different subpopulations of pike have evolved genetically based adaptive differences in growth rates and body size (Tibblin et al., 2015). This indicates that there might be genetic incompatibilities between the locally adapted subpopulations that resulted in the somewhat smaller size of admixed fry in the present study.

Perhaps the most novel conclusion emerging from this study was that the consequences of genetic admixture for a given population combination can vary depending on the origin of the male and female, respectively. Such sex‐specific differences within population combinations were found for both hatching success and fry body length (Figure 4). A possible explanation for this is that antagonistic selection can result in differences in genetic architecture of life history traits between the sexes (Forsman, 1995; Lande, 1980; Rice, 1984; Roff, 1992; Trivers, 1972; Zwoinska et al., 2016), which might contribute to the sex‐specific responses to genetic admixture. Admittedly, our experimental design does not enable us to discard the possibility that the observed differences and effects were mediated to some extent by environmentally induced plasticity and parental effects. Regardless the underlying mechanisms, sex‐specific effects have the potential to affect the ecological and evolutionary dynamics of the populations.

In addition, we found that both maternal and paternal identity influenced all offspring quality measures, indicative of genetic or epigenetic parental effects, or a combination of the two. This conforms with the findings of Siddique et al. (2017) who report that parental identity had a strong influence on embryonic development in pike.

### 4.2 Implications for management and natural dispersal

The pike populations included in this study, despite being located in close proximity to each other in an open system, are genetically separated (Larsson et al., 2015). This fine‐scaled genetic structuring is caused by the homing behavior observed in pike. The results suggested that admixture did not materially impair offspring quality in any of the population combinations, and conversely, none of the populations performed substantially better when admixed. In addition, previous studies have found that the pike populations harbor different local adaptations (e.g., larval traits (Berggren et al., 2016); body size and growth rate (Tibblin et al., 2015); vertebral number (Tibblin, Berggren, et al., 2016); and salinity tolerance (Sunde et al., 2018)). Because of differences in local adaptations, the benefits of such population‐specific adaptations are likely higher in the natural environments than in the laboratory (Forsman, 2014), and dispersal from the environments that individuals are adapted to is likely associated with additional disadvantages to the immigrants in the wild. Taken together, the lack of positive effects of admixture, in combination with the potential costs accruing to migrators and immigrants in novel environments, might explain the persistence of the homing behavior.

Because of the complexity underlying the responses to genetic admixture, it would be practically impossible to investigate genetic architecture, local adaptations, and all potentially important environmental conditions before implementing conservation efforts. Therefore, different proxies (e.g., geographic distance, neutral genetic differentiation, and environmental similarity) have been used to determine which populations to use for translocations and supplementations (Frankham et al., 2011; Garner, Angelone, & Pearman, 2003; Gutiérrez‐Espeleta, Kalinowski, Boyce, & Hedrick, 2000; Liu et al., 2013; Raabová, Münzbergová, & Fischer, 2007; Small, Johnson, Bowman, & Martinez, 2014; Weeks et al., 2011). However, empirical studies investigating the response to admixture in relation to genetic differentiation and/or geographic separation have reported conflicting results (Edmands, 1999; Fenster & Galloway, 2000b; Pélabon, Carlson, Hansen, & Armbruster, 2005; Quilichini, Debussche, & Thompson, 2001; Tortajada et al., 2010; Waser, Price, & Shaw, 2000; Willi & Van Buskirk, 2005), and the suitability of these metrics as proxies to predict the outcome of admixture thus likely differ among species (Edmands, 2002). Our study populations differ in regards to geographic separation and neutral genetic differentiation. However, we did not find any overall effects of genetic admixture on offspring quality in any of the populations combinations. Thus, based on the results from this study, it is not possible to determine if there is any correlation between the degree of geographic distance or genetic differentiation and the response to admixture in pike. Whether and how geographic distance and genetic differentiation influence the direction of the outcome of admixture in pike therefore needs to be further investigated.

It should be emphasized that estimates of neutral genetic diversity and differentiation do not necessarily reflect adaptive diversity (Leinonen et al., 2008; Reed & Frankham, 2001; Tinnert, 2017; Willi, Van Buskirk, & Hoffmann, 2006), and that the outcome of admixture is determined by functional genetic architecture and local adaptations (adaptive diversity) in the involved populations. It is therefore likely that investigations into adaptive divergence could be more informative and suitable for predicting the outcome of genetic admixture than estimates of genetic diversity based on neutral markers (McClelland & Naish, 2007). Future studies should aim at investigating associations between adaptive divergence and the direction of the response to admixture, to increase the understanding of what determines the outcome of admixture.

The finding that the consequences of admixture can be sex‐specific suggests that the response of a given population depends on the sex of the reproducing immigrants, regardless whether the sex effects are mediated by genetic effects, environmental carryover effects, or a combination of genetic and environmental contributions. This adds an additional level to the complexity of mechanisms underlying the response to admixture. Despite this, many studies investigating the effects of admixture treat all offspring from a population combination as a single group, without considering the origin of each parent. By doing this, potential sex‐specific responses may be overlooked. Knowledge about sex‐specific responses can potentially increase the understanding of sex‐dependent antagonistic selection and evolution of biodiversity. From a management or conservation perspective, this can be important to consider when selecting a single or a few individuals for supplementations or translocations, as the decision can impact on the success of such efforts. Sex‐specific responses to admixture can also lead to sex‐biased dispersal and migration. However, it is not known whether dispersal is sex‐biased in these pike populations. Future studies regarding sex‐biased dispersal in relation to sex‐specific effects of genetic admixture may increase the understanding about dispersal behavior and population differentiation.

## 5 CONCLUSIONS

The results of the population combination comparisons indicate that genetic admixture does not have any overall effects on F1 offspring quality in pike. However, these result should be interpreted with caution because previous studies have shown that the direction of the response can differ between different populations (Escobar et al., 2008; Hufford et al., 2012; Sunde & Forsman, 2016; Tortajada et al., 2010), between generations (Huff et al., 2011; Tinnert et al., 2016), and between different environments (see McClelland & Naish, 2007). The lack of an overall effect in the present study is thus not universally transferable to all pike populations.

An interesting finding in our study was that the responses to genetic admixture for some population combinations were sex‐specific, and thus could differ depending on the origin of the male and female respectively. Future studies are necessary to assess the generality of such sex‐specific effects of admixture, and attempts to explore whether sex‐specific responses to admixture are generally associated with sex‐biased dispersal may expand our knowledge and understanding about dispersal behavior and evolution of biodiversity, and aid development of management actions aimed at preservation and revitalization of endangered and declining populations and species.

## CONFLICT OF INTEREST

The authors declare no competing interests.

## AUTHOR CONTRIBUTIONS

J.S., P.T., P.L., and A.F. conceived the study. All authors collected adult fish. J.S. was responsible for the setup and maintenance of the laboratory experiment. A.F. and J.S. analyzed the data. All authors contributed to interpretation of results. J.S. and A.F. drafted the first manuscript. All authors commented on and agreed to the final version of the manuscript.

## DATA ACCESSIBILITY

Data is available in the Dryad Digital Repository: https://doi.org/10.5061/dryad.dd64hf3. Data on performance of admixed and purebred *Esox lucius:* offspring.xlsx. Data on individual body lengths of admixed and purebred *Esox lucius:* fry.xlsx.
